# Whole-Genome Sequencing of Mycobacterium tuberculosis Isolated from Three Hospitals in South Korea

**DOI:** 10.1128/mra.01339-22

**Published:** 2023-01-18

**Authors:** Eon-Min Ko, Hyungjun Kim, Ji-A Jeong, Sungkyoung Lee, Seonghan Kim

**Affiliations:** a Division of Bacterial Disease Research, Center for Infectious Disease Research, National Institute of Infectious Diseases, National Institute of Health, Korea Disease Control and Prevention Agency, Osong, Republic of Korea; b Division of Infectious Disease Control, Bureau of Infectious Disease Policy, Korea Disease Control and Prevention Agency, Osong, Republic of Korea; University of Rochester School of Medicine and Dentistry

## Abstract

Mycobacterium tuberculosis clinical isolates CN118, CN272, CB131, IP004, and IP016 were isolated from three hospitals in South Korea. Here, we report the whole-genome sequences of the five M. tuberculosis clinical isolates to aid the understanding of their genetic diversity.

## ANNOUNCEMENT

Tuberculosis is an infectious disease caused by Mycobacterium tuberculosis. The Global Tuberculosis Report 2021 estimated that 2 billion people were infected with M. tuberculosis, and 1.4 million people died from tuberculosis in 2019 (1). According to the Korea Disease Control and Prevention Agency (KDCA), there were 18,335 new tuberculosis patients in South Korea in 2020, which is considered a major factor threatening public health (2). We performed whole-genome sequencing to confirm the genetic diversity of M. tuberculosis isolated from tuberculosis patients in South Korea.

Study participants were recruited from a tuberculosis cohort from 2016 to 2018. The Institutional Review Board of the KDCA approved this study (2019-04-05-2C-A). All clinical M. tuberculosis isolates (CN118, CN272, CB131, IP004, and IP016) were collected from the sputum of tuberculosis patients at Chungnam National University Hospital (CN118 and CN272), Chungbuk National University Hospital (CB131), and Inje University Ilsan Paik Hospital (IP004 and IP016) in South Korea. Both Löwenstein-Jensen (L-J) medium and the mycobacterial growth indicator tube (MGIT) culture system were used to isolate the clinical isolates from the sputum samples. Positive culture isolates were confirmed by acid-fast bacillus (AFB) staining with Ziehl-Neelsen stain and MPT64 antigen detection using the Bioline TB Ag MPT64 assay (Abbott, Chicago, IL). AFB staining- or MPT64 detection-negative samples were assessed by PCR using the PowerChek MTB/NTM real-time PCR kit (Kogene Biotech, Seoul, South Korea). Spoligotyping was carried out by using a spoligotyping kit (Ocimum Biosolutions, Hyderabad, India) according to the protocol described previously (3).

Genomic DNA was isolated using a *Quick*-DNA fungal/bacterial miniprep kit (Zymo Research, Irvine, CA) following the manufacturer’s instructions. Paired-end (2- by 150-bp) DNA libraries were generated using a TruSeq Nano DNA kit (Illumina, San Diego, CA) and sequenced on an Illumina NovaSeq 6000 platform. Reads generated through sequencing were quality validated and trimmed using Prinseq (v0.20.4) (4). The reads were assembled *de novo* using SPAdes (v3.15.2) (5). Genome annotation was performed using Prokka (v1.14.6) (6). Default parameters were used for all software. The five genomes were all found to be around 4.37 Mbp with a GC content of 65.6% and were in 60 to 76 pieces with *N*_50_ values of ~136,720 to 166,081 after sequencing with ~2 million reads to a sequencing depth of 116 to 148× (Table 1). Annotation features are shown in Table 1.

**TABLE 1:** Assembly statistics of whole-genome sequencing

Parameter	Value for isolate:				
	CN118	CN272	CB131	IP004	IP016
Total no. of reads (bp)	1,910,120	2,260,566	2,127,282	1,994,766	1,769,160
Genome size (bp)	4,369,536	4,366,876	4,366,556	4,376,104	4,371,847
No. of contigs	66	69	75	60	76
GC content (%)	65.6	65.6	65.6	65.6	65.6
Coverage (×)	125	148	140	132	116
*N*_50_ value (bp)	151,905	138,650	136,720	166,081	144,231
No. of coding sequences	4,040	4,033	4,047	4,028	4,043
No. of rRNAs	3	3	3	3	3
No. of tRNAs	52	52	52	53	52
No. of tmRNAs	1	1	1	1	1
Lineage	East Asia	East Asia	East Asia	Euro-American	East Asia

### Data availability.

Accession numbers for the isolates are as follows: CN118, JAOUSP000000000 (GenBank), SAMN31178391 (BioSample), and SRR21820123 (SRA); CN272, JAOUSO000000000 (GenBank), SAMN31178392 (BioSample), and SRR21820122 (SRA); CB131, JAOUSN000000000 (GenBank), SAMN31178393 (BioSample), and SRR21820121 (SRA); IP004, JAOUSM000000000 (GenBank), SAMN31178394 (BioSample), and SRR21820120 (SRA); and IP016, JAOUSL000000000 (GenBank), SAMN31178395 (BioSample), and SRR21820119 (SRA).
